# Academic research careers for medical doctors (ARC-MD): a five-year UC Davis training program to foster the next generation of physician-scientists

**DOI:** 10.1080/10872981.2025.2568575

**Published:** 2025-10-12

**Authors:** Mamta Parikh, Jennifer L. Rosenthal, Luis Fernando Santana, Frederick J. Meyers

**Affiliations:** a Department of Internal Medicine, Division of Hematology Oncology, University of California School of Medicine (UCDSOM) and Associate Director of the ARC-MD Program, Sacramento, USA; b Associate Professor of Pediatrics at the UCDSOM and Associate Director of the ARC-MD Program, Sacramento, USA; c Distinguished Professor and Chair of Physiology and Membrane Biology, Vice Dean for Basic Sciences, and Former Co-Director of the ARC-MD Program, Davis, USA; d Distinguished Professor of Internal Medicine, Division of Hematology and Oncology at the UCDSOM, Associate Director for Education, Training and Career Development of UCD Comprehensive Cancer Center, and Director of the ARC-MD Program, Sacramento, USA

**Keywords:** Longitudinal curriculum, mentorship, research skills, workforce development, physician-scientist

## Abstract

Since the establishment of the Flexnerian model of medical education, single degree (MD) physician-scientists have significantly advanced biomedical research and clinical medicine. However, institutional emphasis has predominantly favoured dual-degree MD/PhD Medical Scientist Training Programs (MSTPs). Recognizing that many successful academic physician-scientists do not hold PhDs, there remains a critical need for structured research training and mentorship targeting single degree students. The University of California Davis School of Medicine developed the Academic Research Careers for Medical Doctors (ARC-MD) program as part of a broader institutional strategy to accelerate innovation, aligning closely with an institutional framework emphasizing thematic breadth and reciprocal interactions between basic and clinical departments. ARC-MD strategically integrates a five-year research-intensive pathway into the traditional four-year MD curriculum, annually enrolling 4−8 students. The program includes an introductory pre-matriculation course, a longitudinal curriculum spanning medical school, focused mentorship fostering interdisciplinary collaboration, a dedicated research year emphasizing reciprocal exchanges between clinical and basic science research, professional identity formation, and financial support through tuition scholarships and stipends. Since its inception in 2019, ARC-MD has enrolled 41 students and graduated two cohorts. Program evaluation surveys were administered in 2024 and 2025 across all five years of training. Student-reported research self-efficacy, measured using the Clinical Research Appraisal Inventory, was lowest among newly matriculating students (pre-program) and increased in subsequent years. Student-reported identity as physician-scientists and student assessments of mentors also showed overall improvement across training levels, though trajectories were not strictly linear.

## Problem

Physician-scientists serve a critical role in bridging the gap between research and clinical practice. At present, traditional medical scientist training programmes (MSTPs) typically involve pursuit of a dual medical and doctoral degree (MD/PhD). Despite growth in MD/PhD programmes, the number of graduates inadequately sustains the physician-scientist workforce [1]. Also, 91% of the physician-scientist workforce does not hold a PhD [2].

Physician-scientists affiliated with an academic institution are more likely to engage in research [2]. Further, an interest in research and identification of a mentor are each independently associated with academic career intentions irrespective of degree [3]. Participation in at least one year of research is also associated with academic career pursuit [4]. Given these associations, a programme cultivating early research exploration and mentorship during training may expand the physician-scientist workforce.

Aligned with our institution's broader strategy to accelerate innovation with reciprocal interactions between basic and clinical research, we sought to recruit students with an interest in research, to a programme focused on accelerating research training while maintaining strong mentorship [5]. We describe the University of California Davis School of Medicine (UCDSOM) Academic Research Careers for Medicine Doctors (ARC-MD) pathway, which aims to train the next generation of physician-scientists.

## Approach to innovation

### Admissions

The ARC-MD admissions process overlaps with existing UCDSOM admissions process for the 4-year MD (4YMD) programme, which has previously been described [6]. Applicants indicate interest in ARC-MD in their secondary application, which is part the 4YMD routine admissions procedures, whereby they briefly describe their envisioned future as a physician-scientist and how they aspire to incorporate research into their career. Selection is based on written application content, the 4YMD programme interviews, and ARC-MD interviews with one ARC-MD director and one ARC-MD student.

In selecting candidates, we consider prior research experience, academic achievements, ability to demonstrate a commitment to research, and potential as an academic physician-scientist. We select cohorts with attention to the stated areas of research interests of students, striving to enroll groups that represent the full translational science spectrum. ARC-MD admits 4−8 students annually, with flexibility to adjust each admissions cycle to meet dynamic needs and facilitate cohorts with diverse research interest. Students receive partial tuition scholarships and a stipend for a dedicated research-intensive year. Philanthropic, National Institute of Health (NIH) TL1 funding, and UCDSOM funding support the programme.

### Curriculum

ARC-MD curriculum objectives, which augment existing SOM clinical curriculum, are to: establish strong mentee-mentor relationships, develop identity as a physician-scientist, enhance research skills, and strengthen team science competencies.

The five-year longitudinal curriculum begins two weeks prior to SOM matriculation with an in-person ARC-MD course consisting of didactics, enrichment activities, and workshops focused on research fundamentals, effective mentorship relationships, and research communication in both oral and written form. Students complete a NIH biosketch and present a research proposal. Dedicated time is allotted to meet with prospective mentors.

**Figure 1.**
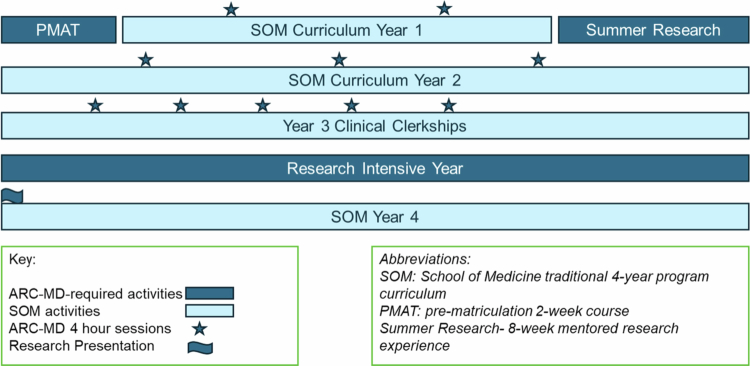
Overview of the ARC-MD five-year longitudinal curriculum.

Subsequently, the ARC-MD curriculum is intercalated with traditional 4YMD coursework (Figure 1). Examples of ARC-MD coursework include advanced skills training with library science staff, research resiliency skills training, and student-driven research presentations to strengthen communication skills, with coursework designed towards building core competencies of clinical and translational research as well as translational scientist characteristics as defined by the NIH [7]. ARC-MD students spend the summer between their first and second year of medical school engaged in an 8-week research project they design with a research mentor, culminating with an oral and written summary of their work. Students may continue to work with this research mentor for their research-intensive year or may select a separate research mentor. ARC-MD directors, who are longitudinal mentors for students, continue to meet with students regularly for career development discussions and to guide selection of appropriate research mentors for a research-intensive year which starts at the end of the third year of medical school.

All students submit a proposal to the UCDSOM Translational Research Training Programme (TL1) for their research-intensive year. The research-intensive year focuses on students individual mentored research and while there is not formal ARC-MD coursework during that time, students participate in TL1 coursework to further hone research skills and identity formation. ARC-MD directors continue to meet with students during the research-intensive year to confirm research progress and to provide support as needed. ARC-MD students present their research at a UCDSOM symposium. Annual programme evaluations led by the UCDSOM Office of Research Evaluation Unit began in 2024. To assess achievement of programme objectives, this evaluation includes student-reported surveys with the Clinical Research Appraisal Inventory (CRAI−12) [8], items assessing professional identity of students as physician-scientists, and assessments of primary research mentors. The evaluation also includes exit focus groups with graduating students. Two members of the SOM Office of Research Evaluation Unit conducted the focus groups. The Evaluation Unit used a guide with probing questions that addressed overall programme experience, research and career skill development, transition to residency, physician-scientist identity, team science, mentoring, and programme support. To protect the anonymity of students, qualitative findings are provided to ARC-MD directors in the form of a summary report.

## Outcomes

Since inception in 2019, we have received around 500 applications a year, with a total of 41 students entering ARC-MD, 14 (34%) reporting first-generation college status (no parent/guardian with a bachelor’s degree). All selected students had some prior research experience. Six students exited the pathway for reasons including transition to the MD/PhD pathway, loss of interest, medical school withdrawal, or difficulty sustaining 4YMD coursework alongside ARC-MD activities. To date, three classes have entered the research-intensive year, supported by NIH TL1 (*n* = 8) or philanthropy (*n* = 3). A summary of student grants, awards, presentations and publications achieved during participation in ARC-MD is included in Table 1. Students who have completed a research-intensive year to date have participated in a variety of research, including basic, clinical and implementation science research. All students in the first two graduating classes successfully matched into residencies in Anaesthesiology (*n* = 1), Internal Medicine (*n* = 5), Family Medicine (*n* = 1), and Psychiatry (*n* = 1) residency programmes.

Programme evaluation surveys were administered in 2024 and 2025 across all five years of training to the active students in ARC-MD, resulting in 41 completed survey packets (100% response rate). Student-reported research self-efficacy, measured using the (CRAI-12) [7], is shown in Table 2. Results assessing student-reported identity as physician-scientists is shown in Table 3, and student assessments of mentors is shown in Table 4.

The Evaluation Unit gathered input from all graduating ARC-MD students in 2024 (*n* = 4) and 2025 (*n* = 3) through virtual focus groups; one student in 2025 who could not attend provided feedback via email. Excerpts from the ‘Overall Experience in ARC-MD’ section of these exit reports are provided in Table 5.

## Limitations and lessons learned

ARC-MD represents a promising national model for physician-scientist training, particularly suited for institutions committed to innovation and interdisciplinary collaboration. The primary challenge involves securing sustainable financial support, as ARC-MD currently benefits significantly from institutional and philanthropic funding. Partial tuition scholarships mitigate financial barriers, but additional strategies may be required to maximize participation.

While ARC-MD currently appeals to students with a pre-existing research interest, future adaptations may expand accessibility to students discovering their research interests mid-training. ARC-MD suits student physician-scientists seeking a contracted pathway over a traditional MSTP, but the programme does not currently offer an additional degree, making the programme potentially less attractive to some applicants. We require students to express an initial interest in academic research careers; inherent in this methodology is exclusion of students who discover research interests during their medical training. This programme may also be attractive to those applying to residencies which look favourably upon research experience, rather our goal of a pre-faculty training track.

As ARC-MD matures, ongoing programme evaluation by the UCDSOM Evaluation Unit will guide refinements to ensure alignment with strategic institutional goals [5]. Limitations of the outcomes presented here include availability of only two years of assessment data. Continued annual data collection will permit future rigorous evaluations of programme impact. Because systematic programme evaluation began in 2024, complete longitudinal data from pre-matriculation through graduation will be available in 2029. While too early to report long-term outcomes given the small cohort that has completed the programme at present, we will track markers of programme success from ARC-MD graduates, including publications, presentations, participation in research during residency and/or fellowship training, academic career positions, and the extent to which research is a component of their effort. These long-term outcomes data will confirm whether this strategy is successfully training an academic workforce and will inform further refinements to the ARC-MD programme.

**Table 1.** ARC-MD programme student achievements.

Achievements	Description
Grants and Scholarships	– 12 of 35 (34%) students received grants and scholars, 4 of which were extramural – Selected examples: American Society of Haematology Opportunities for the Next Generation of Research Scientists (HONOURS); American College of Gastroenterology Medical Student Research Award; American Heart Association Student Scholarship in Cardiovascular Disease
Publications and presentations	– 23 of 35 (66%) of students published manuscripts (83 total) – 15 of 35 (43%) of students published first-author manuscripts (40 total) – Mean (SD) manuscripts per student: 2.3 (3.4) – Median (25%−75% IQR) manuscripts per student: 1 (0−5) – 20 of 35 (57%) of students gave conference presentations (93 total)

Data in table excludes the 8 newest students, as they have been in ARC-MD for less than 3 months.

**Table 2.** Clinical research appraisal inventory (CRAI-12) scores by level of training.

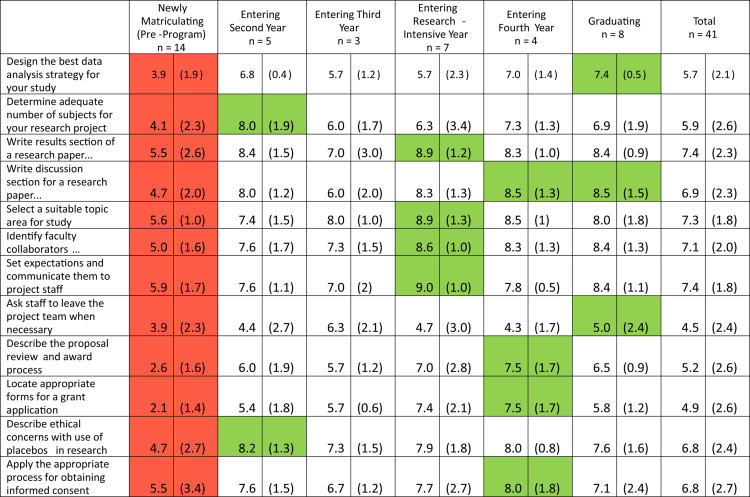

All results reported as mean (standard deviation). CRAI-12 survey questions used the following prompt: ‘We would like to know how confident you are that you can successfully perform these tasks CURRENTLY.’ Response options ranged from 0 (no confidence) to 10 (total confidence). Red cells indicate scores with the lowest point estimate for that item; green cells indicate scores with the highest point estimate for that item.

**Table 3.** Identity as a physician-scientist by level of training.

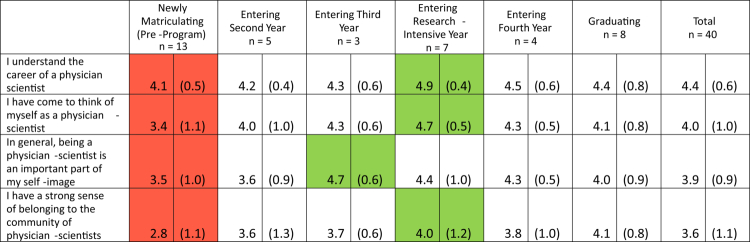

All results reported as mean (standard deviation). The above survey questions used the following prompt: ‘Please rate your level of agreement with the following statements.’ Response options included the following: 1, Strongly disagree | 2, Disagree | 3, Neither agree nor disagree | 4, Agree | 5, Strongly agree. Red cells indicate scores with the lowest point estimate for that item; green cells indicate scores with the highest point estimate for that item.

**Table 4.** Student-reported assessment of primary research mentor.

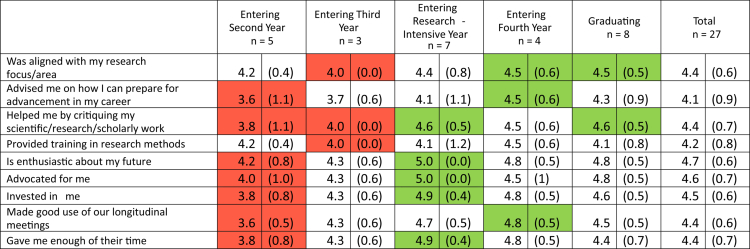

All results reported as mean (standard deviation). The above survey questions used the following prompt: ‘Please indicate below how satisfied you are with the different elements of faculty research mentoring you have experienced as a scholar. My faculty research mentor(s):’ Response options included the following: 1, Strongly disagree | 2, Disagree | 3, Neither agree nor disagree | 4, Agree | 5, Strongly agree. Red cells indicate scores with the lowest point estimate for that item; green cells indicate scores with the highest point estimate for that item.

**Table 5.** ARC-MD programme exiting student focus group summary.

Year	Overall experience in ARC-MD
2024	‘ARC-MD students were asked to provide a few words to describe their overall experience in the programme. They used phrases like ​​​​“learning curve during research year,” “exposure and skillset” (i.e., exposure to academic medicine and what it means to be an academic-physician), “connections to opportunities,” “exposure, growth, expansion,” and “challenging.” Overall, students were thankful for their ARC-MD participation, and it has been significant in their professional development.’
2025	‘ARC-MD students were asked to provide a few words to describe their overall experience in the programme. They used phrases like “educational and productive,” “supportive and encouraging,” and “community and career development”.’
	‘When asked about why they joined the ARC-MD programme, all students noted a previous interest in research and science. Some indicated that ARC-MD was a good balance between a typical MD programme and the MD/PhD programme. For instance, one student said, “just with how long the MD/PhD programme was I didn't really see myself in that. So that's why I picked ARC-MD. You know, I could still stay involved with research, still have a research focus, but it was sort of condensed down into one year”.’

The excerpts above contain the full content of the ‘Overall Experience in ARC-MD’ section from the report prepared by the School of Medicine Office of Research Evaluation Unit.

## Supplementary Material

Supplementary material Reviewer 2 comments 9_24_25
