# Supplementary material for: Academic research careers for medical doctors (ARC-MD): a five-year UC Davis training program to foster the next generation of physician-scientists
Source: Med Educ Online. 2025 Oct 12;30(1):2568575. doi: 10.1080/10872981.2025.2568575 (PMC12519581; doi:10.1080/10872981.2025.2568575)
Supplement: Supplementary material — Reviewer 2 comments 9_24_25 [file ZMEO_A_2568575_SM4084.docx]

| Comment | Response | Section |
| --- | --- | --- |
| The abstract includes “particularly those from educationally and economically adverse backgrounds,” which while is a true statement, seems irrelevant to the current study since not much related to this is discussed other than a statistic about a proportion of participants being first-generation. Additionally, the term “educationally and economically adverse background” is not a commonly used one in the medical education space (i.e., more common terms include disadvantaged backgrounds, underrepresented in medicine, first-generation/low-income in medicine FLGiM) and adverse carries with it a connotation that being first-generation or low-income is a detrimental experience. | We appreciate this comment and agree that this may be less relevant to the current report. As a result, we have opted to remove this statement from the abstract to prevent distraction and certainly to avoid any unintentional detrimental connotation. | Abstract |
| What is the level of financial support offered by the program? Exact dollar amounts are not necessary but a percentage of partial tuition would be helpful for other institutions considering doing something similar, particularly in light of the discussion bringing up how financial feasibility of running this sort of a program is limiting. | We appreciate these questions and understand the interest. Given the dynamic and financially tenuous current climate, we worry about publishing specific percentages should candidates in upcoming application cycles be presented with lower funding levels. | n/a |
| How many applications do they get to the program each year? How much prior research experience do successful applicants have? | We appreciate these questions. While the numbers vary, we receive around 500 applications a year (actual mean = 503), and we have added this information. We have also added a statement noting that all successful applicants have had some prior research experience. In future reports when we have mature data, we will be able to describe in more detail the extent of prior research experience and associated outcomes. | Outcomes |
| The description of the didactics/workshop curriculum during the medical school years is vague. Do these touch on recommended skills outlined by the NIH (link)? What support and teaching are offered by the program during the research-intensive year? | Thank you for this comment. We have provided some examples of coursework, and have added a comment to explain that the curriculum was designed with NIH Clinical and Translational Research Core Competencies. In addition, we note that there is not formal ARC-MD coursework during the research-intensive year but that students do participate in coursework through the TL1 program, and continue to receive intensive mentorship from ARC-MD during that time. We definitely appreciate the suggestion to provide more granular information in future reports. | Approach to Innovation |
| What kind of research are students participating in (clinical vs. basic science vs. translational vs. epidemiology/public health vs. quality improvement)? Certain research types are more amenable to completing projects in the limited time frames of summer and 1 research year, therefore it would be helpful to know what sorts of fields are represented in the work done by trainees, particularly compared to the fact that MD-PhD dual degree programs tend to be more skewed toward basic science/laboratory-based research. | We appreciate these questions and agree that certain research types are more amenable for the compressed timeline presented here. However, in our experience, students have pursued a variety of research projects varying from basic research to public health projects, and we have noted this in our revision. We definitely appreciate these comments as a suggestion of data to present with more granularity in future reports when we have more students who have completed the program. | Outcomes |
| Are the research mentors mostly MDs or PhDs or both? Table 4 provides convincing evidence that trainees felt supported by their advisors throughout their time in the program. However, information about degree-type of mentor could add an interesting layer toward understanding whether it is important toward trainee-reported skills-building and planning a future. | We appreciate this question and comment. We agree that this is an excellent suggestion for future reports. While research mentors in this program have had MD, PhD, or MDPhD degrees, only two classes have completed their research-intensive year at this time, so we believe there will be more value in describing this in future reports. | n/a |
| Interestingly, the confidence in the research-related items does not increase after the research-intensive year (Table 2). Why might this be? It is true that confidence level of >8 out of 10 is already high, but it is curious that it does not increase further (although this may just be due to reluctance of respondents to indicate the extremes in a scale). | We appreciate this question regarding the early results we present. We note a small sample size with potential differences across cohorts, as noted in the statement “outcomes presented here include availability of only two years of assessment data.” However, it is possible that students have greater insight into what they do not know as they progress in the program. Future longitudinal data for students from prematriculation through graduation will be available in 2029 and will provide more insight. | n/a |
| It would be helpful to have more information about M4 outcomes. Do trainees choose to continue working in the lab during M4? What specialties are they pursuing? Do they match? How has participation influenced whether they choose to apply to residency programs that have a research track/component or even formal physician-scientist training programs sponsored by various NIH grant mechanisms? | We appreciate these comments- an earlier submission of this manuscript actually included much of this information about successful match rates and specialties and was inadvertently removed. We have reincorporated it. We definitely plan to collect more data on these outcomes and the influence of our program on residency program selection and success when we have more classes completing the program. | Outcomes |
| Who conducted the focus groups described in Table 5? Did all trainees participate in these focus groups? What questions were used during the focus group? | These questions are appreciated and we have added information on focus group logistics and trainee participation to address this reviewer comment. | Approach to Innovation, Outcomes |
